# Double-Blind Randomized Placebo Controlled Trial Demonstrating Serum Cholesterol Lowering Efficacy of a Smoothie Drink with Added Plant Stanol Esters in an Indonesian Population

**DOI:** 10.1155/2018/4857473

**Published:** 2018-02-04

**Authors:** Lanny Lestiani, Dian Novita Chandra, Kirsi Laitinen, Fransisca Diah Ambarwati, Päivi Kuusisto, Widjaja Lukito

**Affiliations:** ^1^Department of Nutrition, Faculty of Medicine, Universitas Indonesia, Cipto Mangunkusumo General Hospital, Jakarta, Indonesia; ^2^Institute of Biomedicine, University of Turku, 20014 Turku, Finland; ^3^Sanghiang Perkasa-Kalbe Nutritionals, Jakarta, Indonesia; ^4^Raisio Group, P.O. Box 101, 21201 Raisio, Finland; ^5^Human Nutrition Research Center, Indonesian Medical Education and Research Institute, Faculty of Medicine, Universitas Indonesia, Jakarta, Indonesia

## Abstract

Indonesians have a high intake of saturated fats, a key contributing dietary factor to elevated blood cholesterol concentrations. We investigated the cholesterol lowering efficacy of a smoothie drink with 2 grams of plant stanols as esters to lower serum total and LDL-cholesterol concentrations in hypercholesterolemic Indonesian adults. The double-blind randomized placebo controlled parallel design study involved 99 subjects. Fifty subjects received control drink and dietary advice, and 49 subjects received intervention drink (Nutrive Benecol®) and dietary advice. Baseline, midline (week 2), and endline (week 4) assessments were undertaken for clinical, anthropometric, and biochemical variables. Compared to control, the smoothie drink with plant stanols reduced serum LDL-cholesterol concentration by 7.6% (*p* < 0.05) and 9.0% (*p* < 0.05) in two and four weeks, respectively. Serum total cholesterol was reduced by 5.7% (*p* < 0.05 compared to control) in two weeks, and no further reduction was detected after four weeks (5.6%). Compared to baseline habitual diet, LDL-cholesterol was reduced by 9.3% (*p* < 0.05) and 9.8% (*p* < 0.05) in the plant stanol ester group in two and four weeks, respectively. We conclude that consumption of smoothie drink with added plant stanol esters effectively reduces serum total and LDL-cholesterol of hypercholesterolemic Indonesian subjects already in two weeks. Trial is registered as NCT02316808.

## 1. Introduction

Similarly to other countries in Asia-Pacific region, the prevalence of cardiovascular disease in Indonesia is rising [1]. Data from the Indonesian National Household Survey showed that the cause of death from noncommunicable diseases increased from 25 percent in 1980 to 49 percent in 2001 [2]. In a similar pattern, the proportion of deaths arising from cardiovascular diseases in 2001 (26%) tripled since 1986 (9%) [2] and has continued to rise. Recent data indicates that the cardiovascular diseases are already the leading cause of death in Indonesia, accounting for 37% of all mortality [3]. In Indonesia the major risk factors for cardiovascular diseases are elevated blood pressure, elevated blood cholesterol, and cigarette smoking [1]. Prevalence of elevated (>200 mg/dl) total cholesterol is estimated to be 40% in women and 30% in men [1]. Ideally, strategies to reduce the incidence of cardiovascular diseases should be targeted at primary prevention, especially through dietary habits [4]. Furthermore, healthy dietary practices contribute to reduced morbidity and mortality rates also for secondary prevention [5].

Initial maneuvers to manage dyslipidemia should be through a dietary approach with a particular focus on improving the quality of ingested dietary fat [6]. Therapeutic lifestyle changes are however often considered burdensome and call for easily applicable means for dietary modification [7]. The development of functional foods, with specific functional properties additive to normal nutritional value, should be pursued. Plant stanols incorporated in foods as esters represent an important functional food substance with lipid lowering properties. Vigorous scientific evidence pertaining the total and LDL-cholesterol lowering properties of plant stanol esters has led to their adoption as a target for lipid lowering modalities by several international bodies [8].

While many studies demonstrating the lipid lowering effects of plant stanol esters have been carried out on Caucasian subjects [9], the evidence is scarce for non-Caucasians, including Asians, and needs to be confirmed due to potential cultural differences—through different food habits and practices. The mainstream Indonesians are known as a population with a high intake of saturated fats [10, 11], the key contributory dietary component to high total cholesterol and LDL-cholesterol concentrations [7, 12]. Therefore, the present study aimed to demonstrate the lipid lowering effects of plant stanol esters in an Indonesian population.

## 2. Materials and Methods

### 2.1. Study Design and Subjects

This double-blind randomized controlled parallel design study was conducted at the Department of Nutrition, Faculty of Medicine, University of Indonesia. The study involved 99 subjects (44 male and 55 female), aged 24–68 (mean age 47) years, who met the following entry criteria: total cholesterol concentration ≥ 200 mg/dL and <300 mg/dL and/or LDL-C concentration ≥ 130 mg/dL and <240 mg/dL, willingness to consume control or plant stanol ester smoothie drinks two bottles per day immediately after a meal for four weeks, being reachable by phone, and willingness to declare written informed consent, and agreement in accordance with data protective stipulations and readiness to participate in the trial and to adhere to the study conditions. Pregnant women, obese (BMI > 30 kg/m^2^), diabetic subjects or subjects with random plasma glucose concentration >200 mg/dL, subjects with hyperthyroidism, a history of metabolic, endocrine and kidney disorders, and acute or chronic severe diseases possibly interfering with the evaluation of the outcome of the clinical trial (such as AIDS, tumor diseases, malignant hypertension, and cardiac insufficiency according to NYHA III-IV), and subjects who were taking lipid lowering medication or other medications likely to affect lipid metabolism were excluded from the study.

Prior to commencement of the screening, the study was explained to each subject and a written informed consent was signed. The study protocol was approved by the Medical Ethics Committee of the Faculty of Medicine, Universitas Indonesia, Jakarta, Indonesia. The study conforms to the provisions of the Declaration of Helsinki in 1995, as revised in Edinburgh in 2000.

**Figure 1:**
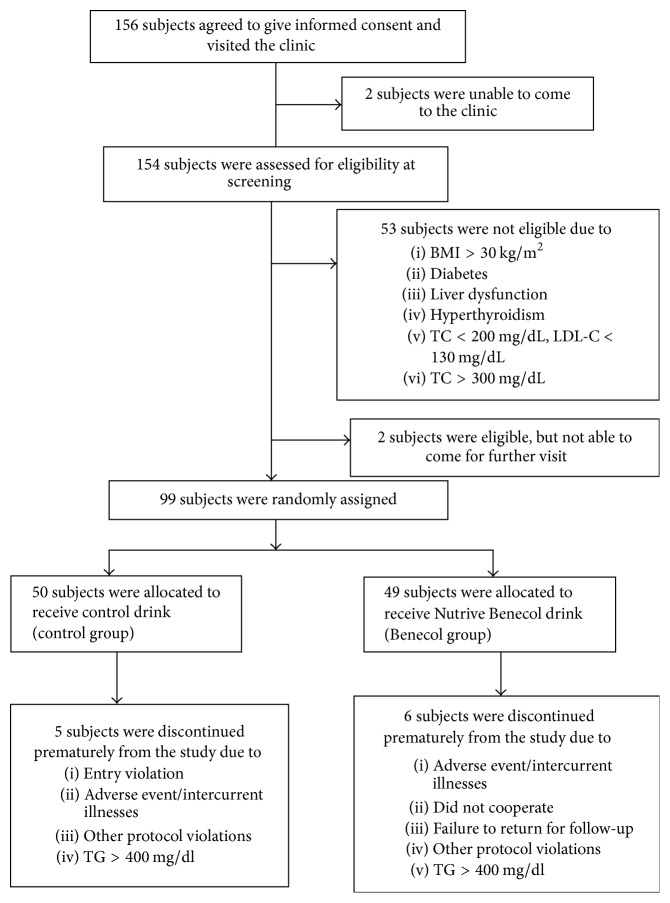
Study flow chart. BMI, body mass index; TC, total cholesterol; LDL-C, low-density lipoprotein cholesterol; HDL-C, high-density lipoprotein cholesterol.

A total of 156 subjects indicated their interest to participate in the study. They were invited to visit the clinic for screening of the eligibility.  Figure 1 depicts the study flow. 99 subjects who met the entry criteria at screening were randomly allocated in a double-blind manner to receive either control (*n* = 50) or plant stanol ester (*n* = 49) smoothie drinks. Simple randomization was used. Three subjects in the control group and five subjects in the plant stanol ester group discontinued the study. Of the subjects who completed the intervention, two in the control group and one in the plant stanol ester group were excluded from the statistical analyses because of triglyceride levels >400 mg/dl at one or several sampling points, making LDL-cholesterol determination by Friedewald formula inappropriate [13]. An overall of 88 subjects were included in the statistical analyses.

The study included four visits to the study clinic, including a screening visit.  Table 1 summarizes the overall procedures undertaken on each subject at screening (week −2), baseline (week 0), midline (week 2), and endline (week 4) phases. Clinic visits within an interval of two days before or after the appointment date were tolerated.

### 2.2. Clinical Examination

General physical examination was undertaken to obtain information on vital signs and to detect physical and neurological abnormalities. Sitting blood pressure was measured using a sphygmomanometer with standard procedure. Subjects were seated for at least five minutes before the actual measurement was taken. Two measurements were taken with a 5-minute interval, and the average value was used in the analyses. Equally, the mean of two measurements for anthropometric indicators was used in analyses. Body weight was measured in kilograms to the nearest 0.1 kg, with light clothes on, using a beam scale Tanita (Tanita, Japan). Stature was measured in centimeters to the nearest 0.1 cm, in a standing position with socks and shoes removed, using a microtoise. Abdominal circumference was measured in centimeters to the nearest 0.1 cm, using a flexible nonelastic tape (Roche, Switzerland), the measurement taken at the midway region between the lowest rib margin and the iliac crest, in standing position with abdomen relaxed, feet together, and weight equally divided over both legs. Body mass index (BMI) was calculated by dividing body weight in kg by height in squared meter.

### 2.3. Biochemical Assessments

Blood specimens were collected in the morning between 7:00 till 10:00 am after an overnight fast of 12 hours, and plasma and serum were separated immediately. All biochemical analyses were performed at certified Prodia Clinical Laboratory (International Standard Operation (ISO) accreditation version 2000:9000 since 1999 and ISO version 15189 since 2007, Jakarta, Indonesia). The determinations were done immediately after the blood separation process. Biochemical parameters like alanine amino transferase (ALT), aspartate amino transferase (AST), creatinine, total and direct bilirubin, blood urea, and fasting blood glucose (FBG) were analysed by routine standardized methods. Serum triglycerides (TG), total cholesterol (TC), and high-density lipoprotein cholesterol (HDL-C) were measured using standard enzymatic methods and LDL-cholesterol (LDL-C) concentration was calculated by using the Friedewald formula [13].

### 2.4. Dietary Advice

At baseline, two trained dietitians instructed the subjects about the diet necessary for healthy eating as recommended by the Indonesian Heart Foundation [14]. Dietary counseling occurred on an individual basis. To the subjects, the dietitians emphasized the need to adhere to dietary advice. Both groups received similar dietary advice. Adherence to the recommendations, or dietary intake, was not assessed during the intervention.

### 2.5. Test Food Product and Compliance

The test food product was a smoothie drink (Nutrive Benecol) produced by PT Sanghiang Perkasa, Jakarta, Indonesia (known as Kalbe Nutritionals). The subjects consumed two bottles of the drink/day, every morning and evening immediately after the main meals. Each 100 mL bottle contained 1 g plant stanols as esters, equivalent to 1.7 g plant stanol ester; thus the total daily dose was 2 g plant stanols as esters. A control drink with similar taste, flavor, and color but without plant stanol esters was produced by PT Sanghiang Perkasa, Jakarta, Indonesia (known as Kalbe Nutritionals) and consumed similarly to the plant stanol ester smoothie drink. The nutritional compositions of the control and plant stanol ester smoothie drinks are shown in Table 2. To ensure blinding of the intervention smoothie drinks, both were packed using the same materials and labeled with codes. Detailed explanation on how to consume the drink (verbal and written) was given by the investigators and trained research assistants. Subjects were also enrolled in a telephone-based support program, which provided regular motivational calls on weekly basis by the investigators throughout the study to improve compliance.

Assessment of compliance was undertaken by asking subjects to return all bottles received at the previous visit to the research clinic. The returned bottles were counted and checked and the left-over volume was recorded by the research assistants. Subjects were considered fully compliant if they consumed 200 mL of either control or plant stanol ester smoothie drink daily, which was equivalent to 5,600 mL for four weeks. If the subject was taking less than 80% of the expected volume (i.e., less than 4,480 ml), the subject was warned about his/her compliance. If the subject was taking less than 60% of the expected volume, the subject was excluded from the study. No subject replacement was undertaken during the course of the study.

### 2.6. Statistical Analyses

A sample size of total 39 randomized subjects in each group was determined in order to detect 10% difference in LDL-cholesterol between the two groups with 80% power and 0.05 two-sided significance. To take into account possible drop out, 99 patients were randomized.

The primary outcome variable in this study was the percentage change (%) in calculated serum LDL-C from baseline up to 4 weeks smoothie drink consumption in the plant stanol ester group compared to the control group. The secondary outcome variables were the percentage changes (%) in serum TC, TG, and HDL-C.

Data analysis was done using SPSS 23.0 statistical analysis software for Windows (SPSS Inc., Chicago, IL, USA). Distributions of continuous variables were assessed for normality using the Kolmogorov-Smirnov test and by visually examining histograms. The plant stanol ester group was compared with the control group with respect to the primary and secondary outcome variables, vital signs, clinical laboratory tests, adverse events, and compliance. For continuous variables, the analyses were performed by independent samples Student's* t*-test, with 95% confidence intervals. The more robust Student's* t*-test was chosen instead of a nonparametric test also for variables having a minor deviation from normality. However, whenever the continuous variables were not normally distributed, the significance levels were confirmed by Mann–Whitney test. Only the* t*-test results are reported. Distributions of categorical variables (gender, smoking habits, and menopausal status of women) between treatment groups were analysed using a chi-square test. Changes in serum lipids from baseline within a group were assessed by using paired samples* t*-test. Whenever the variables were not normally distributed, the significance levels were also confirmed by Wilcoxon signed-rank test. The* t*-test results are reported. In the statistical analyses, a *p* value less than 0.05 was considered as statistically significant.

## 3. Results

### 3.1. Subject Characteristics and Compliance

Baseline characteristics of the subjects by the intervention group are described in Tables 3 and 4 (serum lipids). The groups were comparable in gender, age, and anthropometric and clinical variables. A higher percentage of women at reproductive age existed in the control group, but this difference was not statistically significant (NS). No differences in smoking habits were found between control and plant stanol ester groups.

In the control group, body weight increased by 0.6 kg (*p* < 0.05) during the four weeks of intervention, whereas it remained unchanged in the plant stanol ester group. However, there were no differences in body weight or in other anthropometric indicators between control and plant stanol ester group at the endline visit, after the four-week intervention period (results not shown). No difference between the groups was detected in fasting blood glucose levels at the endline visit either. Eleven subjects in the control (24%) and 13 subjects (30%) in plant stanol ester group reported adverse events during the study. These included gastrointestinal symptoms like changes in stool consistency, epigastric discomfort, bloating, frequent flatus, dyspepsia, and diarrhea. However, these adverse events were generally mild and transient, and no difference between the study groups was detected.

The compliance, measured as the left-over volume of the drink in returned bottles, was on high level and there were no differences between the groups. In the control group, subjects consumed on average 98.6% (5,524 ml) and in the plant stanol ester group 97.4% (5,457 ml) of the provided drink. In the control group 93% of the subjects and in the plant stanol ester group 81% of the subjects consumed at least 5,400 ml (96%) of the provided 5,600 ml of the drink. None of the subjects was excluded from the statistical analyses because of compliance less than 60%.

### 3.2. Changes in Serum Lipids due to Intervention

Compared to the control group, serum LDL-C concentration was reduced by 7.6% (*p* < 0.05) and 9.0% (*p* < 0.05) in the plant stanol ester group after two and four weeks of intervention, respectively (Table 4). Serum total cholesterol concentration was reduced by 5.7% after two weeks, and there was no further reduction after four weeks (5.6%) compared to the control group (Table 4). Serum TG and HDL-C concentrations did not differ between the groups at any time point.

Reduction of serum LDL-C concentration from baseline to two weeks (9.3%) and from baseline to four weeks (9.8%) was statistically significant (*p* < 0.05) in the plant stanol ester group (Table 4). Compared to baseline, also serum TC was significantly reduced (5.8%, *p* < 0.05) after two weeks. No statistically significant further reductions were discovered after four weeks of intervention (5.9%). In the control group, no reduction in serum TC (−0.2% and −0.4% after two and four weeks, respectively) or LDL-C (−1.7% and −0.8% after two and four weeks, respectively) was detected relative to the baseline measurement (Table 4).

## 4. Discussion

Here we demonstrated that consumption of a smoothie drink with added plant stanol esters effectively reduces serum total and LDL-cholesterol in hypercholesterolemic Indonesian subjects. The cholesterol lowering effect was seen already in two weeks and was sustained for four weeks, that is, for the period of plant stanol ester consumption.

This study was designed in an attempt to demonstrate the cholesterol lowering efficacy of plant stanols as esters, incorporated into smoothie drink in Indonesian subjects. Recognizing the fact that the cholesterol lowering efficacy of plant stanol ester has been confirmed in more than 70 clinical studies [9], the current study has at least three distinctive characteristics. Firstly, the study was conducted in Indonesian adult subjects, who have different food habits and practices from subjects in the previous studies. For example, the intake of saturated fatty acids in Indonesia is one of the highest in the world [11]. Secondly, both the control group and the plant stanol ester group subjects received individual dietary advice according to the recommendations of Indonesian Heart Foundation [14] at their baseline visit. Thus this study compared the effects of 2 g plant stanols and dietary advice both to the effects of dietary advice (control group) and to the habitual diet of the subjects (baseline). Thirdly, a smoothie drink with plant stanol ester was applied, while in most of the other clinical studies plant stanol ester has been incorporated in mayonnaise, regular or low-fat-spreads, or drinkable or spoonable yoghurts [15–23].

Plant stanol ester is one of the most established cholesterol lowering functional food ingredients. Its LDL-cholesterol lowering efficacy has been demonstrated as a part of typical Western diet [16] and also as a part of low saturated fat and low-cholesterol diets [17, 18, 21]. While most of the studies have been conducted in European populations, studies in Asian populations are scarce [24–28]. In addition to different genetic backgrounds, there are also differences in dietary patterns and food preferences in different populations. Traditionally, a dominant role of rice and a relatively low level of protein intake have been characteristic for Indonesian diets [29]. Compared to typical Western diets, the share of meat and dairy products of the total calories is still small, for example, 2.6 E-% and 0.7 E-% in 2002, respectively (calorie satisfied urban households) [29]. However, the total fat intake (as E-%) is comparable to Western countries, and the intake of saturated fat (as E-%) exceeds that of most Western countries [10, 11]. In a review of data from 40 countries, Indonesian average intake of saturated fatty acids, 21 E-%, was the highest among the countries studied and for example almost double to the intake in USA [10]. In a study of Hatma et al. [30], the intake of saturated fatty acids varied from 19.8 to 23.5 E-% among four diverse ethnic groups in Indonesia. Important fat sources in Indonesia are palm oil, palm kernel oil, and coconuts [31]. The current study is the first one to demonstrate the LDL-cholesterol lowering efficacy of plant stanol ester in Indonesian population. The efficacy was found to be comparable to what has been found with the plant stanol dose of 2 grams in other populations [9, 32, 33].

In the current study, both the control group and the plant stanol ester group subjects received similar dietary advice according to the recommendations of Indonesian Heart Foundation [14]. The counseling occurred on individual basis at the baseline visit. All subjects were also enrolled in a telephone-based support program that provided weekly motivational phone calls by the investigators. Adherence to the dietary recommendations was not measured. This resembles a real-life situation at clinic, where patients are encouraged to make healthy dietary choices, but their adherence to the advised diet is usually not monitored. In the control group, receiving dietary advice and a control drink, serum TC and LDL-C did not change significantly from the baseline during the four-week intervention. On the contrary, plant stanols effectively lowered both serum TC and LDL-C already in two weeks, and the effect was sustained as long as plant stanol ester was consumed, that is, for four weeks. The findings are in line with the study of Hallikainen and Uusitupa [18], in which plant stanol ester as a part of a low-fat, low-cholesterol diet reduced LDL-C effectively when compared both to the effects of the low-fat, low-cholesterol diet alone, and to the baseline high-fat diet. In another study [34], where subjects were on a hypolipidemic diet already at baseline, LDL-C reduction was faster and greater with plant stanol ester than with Mediterranean diet. The present study confirms the fast effects of plant stanol ester.

Recent studies demonstrate clear dose-response effect for plant stanol ester [9, 35], although data available for higher than 4 g doses are limited [33]. The European Food Safety Authority Panel on Dietetic Products, Nutrition and Allergies has concluded that plant stanols (provided as plant stanol ester) at a daily intake of 1.5–2.4 grams lower LDL-cholesterol by 7–10% and at a daily intake of 2.5–3 grams lower LDL-cholesterol by 10–12.5% [36]. High compliance with the targeted dose thus benefits the subjects in increasing the efficacy of plant stanol ester consumption. Targeted daily dose of plant stanols in the current study was 2 grams/day. The compliance in consuming the smoothie drink was good; 97.4% of the drink volume in plant stanol ester group and 98.6% in the control group were consumed. Thus the actual average intake of plant stanols was 1.9 g/day in the plant stanol ester group. The serum LDL-C reduction obtained is well in line with the expected calculated effect for this dose [32]. Subject self-reported compliance is often used in clinical studies with plant sterols and plant stanols, but description of the compliance quantitatively by the investigators is usually not available.

In most of the previous studies, plant stanol ester has been incorporated in mayonnaise, regular or low-fat-spreads, or drinkable or spoonable yoghurts [15–23]. Apart from drinkable yoghurts, other drinks have seldom been used as a matrix for plant stanol ester [24, 26, 37]. One could speculate that there may be no differences in plant stanol ester efficacy when incorporated in different product matrices. However, a meta-analysis of Demonty et al. [38] showed that phytosterols in doses >2 g/d had a larger cholesterol lowering effect when incorporated in solid foods rather than in liquid foods. It is thus important to confirm the efficacy of plant stanols in different product formats. The smoothie drink of the current study contained dairy protein, but was not fermented unlike the yoghurt drinks of previous studies. The current study thus added information about the cholesterol lowering efficacy of plant stanol ester in nonfermented drinks. Plant stanols was showed to be efficient also in this product format.

## 5. Conclusions

Our findings suggest that consumption of a smoothie drink with added plant stanols (2 grams) as esters effectively reduces serum TC and LDL-C concentrations in hypercholesterolemic Indonesian subjects. Our study, along with the previous studies [24–28], demonstrates that plant stanol ester is an effective means in cholesterol lowering also in Asian populations and as part of Asian diets, but in a new food format. The full effect in reducing the cholesterol levels was achieved already in two weeks and maintained with continued consumption. The findings of our study may be of importance when considering dietary means to reverse the increasing prevalence of cardiovascular morbidity in non-Western cultures [4].

## Figures and Tables

**Table 1:** Schedule of assessments.

	Week			
	−2 (screening)	0	2	4
Informed consent	**X**			
Medical history	**X**			
Physical examination	**X**	**X**		**X**
Anthropometric measurement	**X**	**X**		**X**
Biochemical tests	**X**	**X**	**X**	**X**
Healthy diet advice		**X**		
Adverse events			**X**	**X**
Concurrent medications		**X**		**X**
Monitoring through phone calls			**X**	**X**
Compliance assessment			**X**	**X**

**Table 2:** Nutritional composition of control and plant stanol ester smoothie drinks^*∗*^.

	Control drink	Plant stanol ester drink
*Trade name*	-	Nutrive Benecol
*Form*	Smoothie drink	Smoothie drink
*Composition*		
Serving size 100 mL^†^ containing		
Energy (kcal)	80	80
Carbohydrate (g)	15	15
Protein (g)	1	1
Total fat (g)	2	2
Plant stanol ester (g)		1.7^‡^

^*∗*^Ingredients: water, sweeteners, plant stanol ester (in plant stanol ester drink), skim milk powder, stabilizer, juice concentrate, emulsifier, flavoring, and acidity regulator. ^†^Two servings consumed per day. ^‡^Equivalent to 1 g plant stanols.

**Table 3:** Baseline demographic, clinical, and biochemical characteristics of the subjects in the control and plant stanol ester groups.

Variable	Control group *n* = 45	Plant stanol ester group *n* = 43
Sex, male (*n*, %)	22 (48.9)	16 (37.2)
Age (years)	47.7 ± 11.6	47.4 ± 10.9
Body weight (kg)	61.1 ± 11.0	61.2 ± 10.5
Height (cm)	159.9 ± 8.1	158.5 ± 9.4
BMI (kg/m^2^)	23.8 ± 3.2	24.3 ± 2.8
Abdominal circumference (cm)	85.1 ± 8.6	86.0 ± 8.9
Premenopausal of the women (*n*, %)	14 (60.9)	14 (51.9)
Smoking habits, smokers (*n*, %)	5 (11.1)	7 (16.3)
Systolic blood pressure (mmHg)	110 (103–123)	118 (110–120)
Diastolic blood pressure (mmHg)	70 (68–80)	70 (70–80)
Fasting blood glucose (mg/dL)	94 (88–98)	95 (91–99)

Data are presented as mean ± SD or median (quartile 1–quartile 3). Differences between control and plant stanol ester groups were not statistically significant (independent samples *t*-test, chi-square test).

**Table 4:** Serum lipid concentrations (mg/dL) at baseline (0 weeks) and at 2 and 4 weeks after initiation of the intervention and changes from the baseline in the control and plant stanol ester groups.

Variable	Control group (*n* = 45)	Plant stanol ester group (*n* = 43)	Mean difference between groups (95% CI)
Total cholesterol			
0 weeks (mg/dL)	229.6 ± 27.0	226.3 ± 32.5	−3.3 (−15.9 to 9.3)
2 weeks (mg/dL)	228.5 ± 26.0	212.5 ± 33.3^*∗*†^	−16.0 (−28.6 to −3.3)
4 weeks (mg/dL)	228.0 ± 26.6	212.5 ± 31.9^*∗*†^	−15.6 (−28.0 to −3.1)
% change 2–0 weeks	−0.2 ± 7.7	−5.8 ± 9.0^*∗*^	−5.7 (−9.2 to −2.1)
% change 4–0 weeks	−0.4 ± 8.4	−5.9 ± 7.4^*∗*^	−5.6 (−8.9 to −2.2)
LDL-cholesterol			
0 weeks (mg/dL)	155.0 ± 26.2	151.2 ± 32.0	−3.8 (−16.2 to 8.6)
2 weeks (mg/dL)	151.4 ± 25.5	136.0 ± 29.0^*∗*†^	−15.4 (−27.0 to −3.9)
4 weeks (mg/dL)	152.4 ± 23.1	135.9 ± 30.1^*∗*†^	−16.5 (−27.9 to −5.2)
% change 2–0 weeks	−1.7 ± 12.2	−9.3 ± 13.0^*∗*^	−7.6 (−12.9 to −2.2)
% change 4–0 weeks	−0.8 ± 11.2	−9.8 ± 9.6^*∗*^	−9.0 (−13.4 to −4.6)
HDL-cholesterol			
0 weeks (mg/dL)	50.1 ± 10.6	49.7 ± 9.7	−0.4 (−4.8 to 3.9)
2 weeks (mg/dL)	50.5 ± 11.6	48.4 ± 9.5	−2.1 (−6.6 to 2.4)
4 weeks (mg/dL)	49.8 ± 10.5	49.7 ± 11.1	−0.1 (−4.7 to 4.5)
% change 2–0 weeks	0.9 ± 8.7	−2.0 ± 9.8	−2.9 (−6.8 to 1.0)
% change 4–0 weeks	−0.4 ± 10.8	0.2 ± 11.2	0.5 (−4.1 to 5.2)
Triglycerides			
0 weeks (mg/dL)	122.6 ± 53.3	127.2 ± 67.6	4.6 (−21.1 to 30.4)
2 weeks (mg/dL)	132.8 ± 69.2	140.8 ± 67.3^†^	8.0 (−20.9 to 37.0)
4 weeks (mg/dL)	129.1 ± 56.8	134.4 ± 70.4	5.3 (−21.8 to 32.3)
% change 2–0 weeks	8.9 ± 30.6	16.3 ± 35.5	7.4 (−6.6 to 21.4)
% change 4–0 weeks	11.0 ± 35.1	11.0 ± 33.6	−0.1 (−14.6 to 14.5)

Data are presented as mean ± SD. ^*∗*^Significant difference between control and plant stanol ester groups (*p* < 0.05, independent samples *t*-test). ^†^Significant difference compared to the baseline (*p* < 0.05, paired samples *t*-test).
